# Urbanization alters the relative importance of local and landscape factors affecting plant communities in the Tokyo megacity

**DOI:** 10.1002/ece3.70006

**Published:** 2024-08-29

**Authors:** Yuki Iwachido, Kei Uchida, Takehiro Sasaki

**Affiliations:** ^1^ Graduate School of Environment and Information Sciences Yokohama National University Yokohama Kanagawa Japan; ^2^ Institute for Sustainable Agro‐Ecosystem Services The University of Tokyo Nishitokyo Tokyo Japan; ^3^ Faculty of Environmental Studies Tokyo City University Yokohama Kanagawa Japan; ^4^ Institute for Multidisciplinary Sciences Yokohama National University Yokohama Kanagawa Japan

**Keywords:** landscape factor, local factor, Moran's eigenvector map, plant community, urban green space, variation partitioning

## Abstract

Plant communities are impacted by local factors (related to environmental filtering) and landscape factors (related to dispersal limitation). While many studies have shown that the relative importance of these factors in understanding plant community dynamics due to urbanization, little is known about how they are altered by urbanization—a significant threat to biodiversity. This study evaluates the relative importance of local environmental (local factors), landscape, and spatial (landscape factors) variables that influence plant communities in 34 urban green spaces comprising two different habitats (forests and grasslands) along the urban–rural gradients in the Tokyo megacity, Japan. To continuously assess the relative importance of each factor along the urban–rural gradients, we extracted 1000 landscapes within a certain range that contained several sites. Subsequently, the relative importance of each factor and urbanization rate (proportion of artificial built‐up area) were estimated for each landscape. Our study found that the relative importance of both local and landscape factors decreased, while that of local factor for native species in forest habitats and that of landscape factors for native species in grassland habitats increased. Collectively, these findings suggest that city size and habitat characteristics must be considered when predicting changes in plant communities caused by urbanization.

## Graphical abstract

We investigated local and landscape factors driving plant communities in the Tokyo metropolitan area. The results revealed that the drivers of plant communities change along the urban–rural gradient.

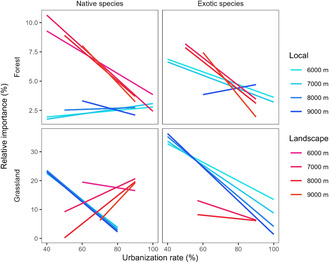

## 1 INTRODUCTION

Biological communities are driven by local factors (e.g., environmental filtering and species interactions) and landscape factors (e.g., dispersal limitation) (Leibold et al., 2004; Vellend, 2010). Previous studies have shown that local and landscape factors do not exert independent effects but rather act simultaneously on biological communities (Chase & Myers, 2011; Leibold et al., 2004). Consequently, delineating their relative importance is a fundamental theme in community ecology, although achieving consensus on their relative importance remains challenging. One reason for this difficulty is that their relative importance could vary with environmental changes. For instance, land use change from intact forest to plantation forest has been shown to shift the relative importance of factors driving mammal communities from landscape factors to local factors (Wearn et al., 2019). Conversely, environmental changes due to wildfires did not alter the relative importance of factors influencing tree communities (Myers et al., 2015). Thus, the variation in their relative importance with environmental change could be context‐depends. However, there is a paucity of data on how the relative importance of each factor varies with urbanization, a process that represents a primary cause of habitat fragmentation and degradation (Grimm et al., 2008), leading to the decline and local extinction of many flora and fauna (Duncan et al., 2011; Hahs et al., 2009; Li et al., 2022; Seto et al., 2012; Simkin et al., 2022). Furthermore, given that more than half of the world's population currently relies on the ecological functions and services provided by urban ecosystems (United Nations, 2019), understanding changes in the relative importance of these factors with urbanization can help predict changes in biodiversity due to urbanization and lead to effective ecosystem management.

Plants are the most studied taxa in terms of the effects of urbanization (Rega‐Brodsky et al., 2022), while urbanization‐related changes in plant communities cannot be accurately predicted. This might be the consequence of a previous oversimplification of the environmental changes caused by urbanization that does not account for the diversity within developed areas, for example, between urban and rural settings (McDonnell & Hahs, 2008; Shochat et al., 2006). Moreover, plants are not only the foundation of urban biodiversity but also provide ecosystem services to more than half of the global population (Aronson et al., 2017; Schwarz et al., 2017). Therefore, it is crucial to accurately predict changes in plant communities due to urbanization, which requires understanding the factors that drive them.

Urbanization has fragmented habitats and reduced connectivity among habitats (McDonnell & Hahs, 2008; McKinney, 2002; Williams et al., 2009). Consequently, urban centers are dominated by specific urban‐adapted species, such as high dispersal species, while losing spatial variation in plant species composition (biotic homogenization) (McKinney, 2006; Palma et al., 2017; Smart et al., 2006; Williams et al., 2015). Thus, habitat fragmentation due to urbanization may represent a weaker influence on dispersal limitation. In addition, urbanization increases environmental heterogeneity within and among habitats (Aronson et al., 2017; Nielsen et al., 2014; Zhou et al., 2017). That is, within urban areas, some of the remnant green spaces have been converted into other green spaces, such as parks and developed green spaces to prevent biodiversity loss; hence, various green spaces exist with different land uses and histories. The increasing environmental heterogeneity associated with urbanization may represent a strong environmental filtering influence that drives plant communities, a concept that has been demonstrated in soil bacteria (Zhang et al., 2020) and aquatic communities (He et al., 2022).

Moreover, urban plant communities can differ in factors driven by native and exotic species (Lopez et al., 2018). Exotic species richness generally increases with urbanization (Aronson et al., 2015; Cadotte et al., 2017; McKinney, 2006) as most have greater dispersal ability than native species. Thus, dispersal limitation may not affect exotic species in urban centers. Conversely, given that native species richness decreases with urbanization, the impact of dispersal limitation may be smaller; however, its impact may be larger than that for exotic species. Also, exotic species are likely to invade newly developed green spaces with low plant diversity and degraded habitats (Cadotte et al., 2017). Given that exotic species can establish themselves in urban center regardless of habitat environments, the impact of environmental filtering on exotic species may decrease with urbanization, opposite to native species.

In this study, we assessed the relative importance of the local and landscape factors that affect plant species communities along an urban–rural gradient in the Tokyo metropolitan area of Japan. To estimate the relative importance of the two factors, we measured the local habitat environment as the local factor and habitat size, surrounding land use, and spatial distribution of habitat as the landscape factor. Moreover, we examined the relationships between the relative importance of these factors and the proportion of artificial built‐up areas around habitats (hereafter urbanization rate) using a linear mixed model (LMM). Specifically, the following hypotheses were tested:

1. While the relative importance of the local factor increases, that of the landscape factor decreases with urbanization.
2. The relationship between the relative importance of each factor and urbanization differs for native and exotic species.

## 2 MATERIALS AND METHODS

### 2.1 Study area

**FIGURE 1:**
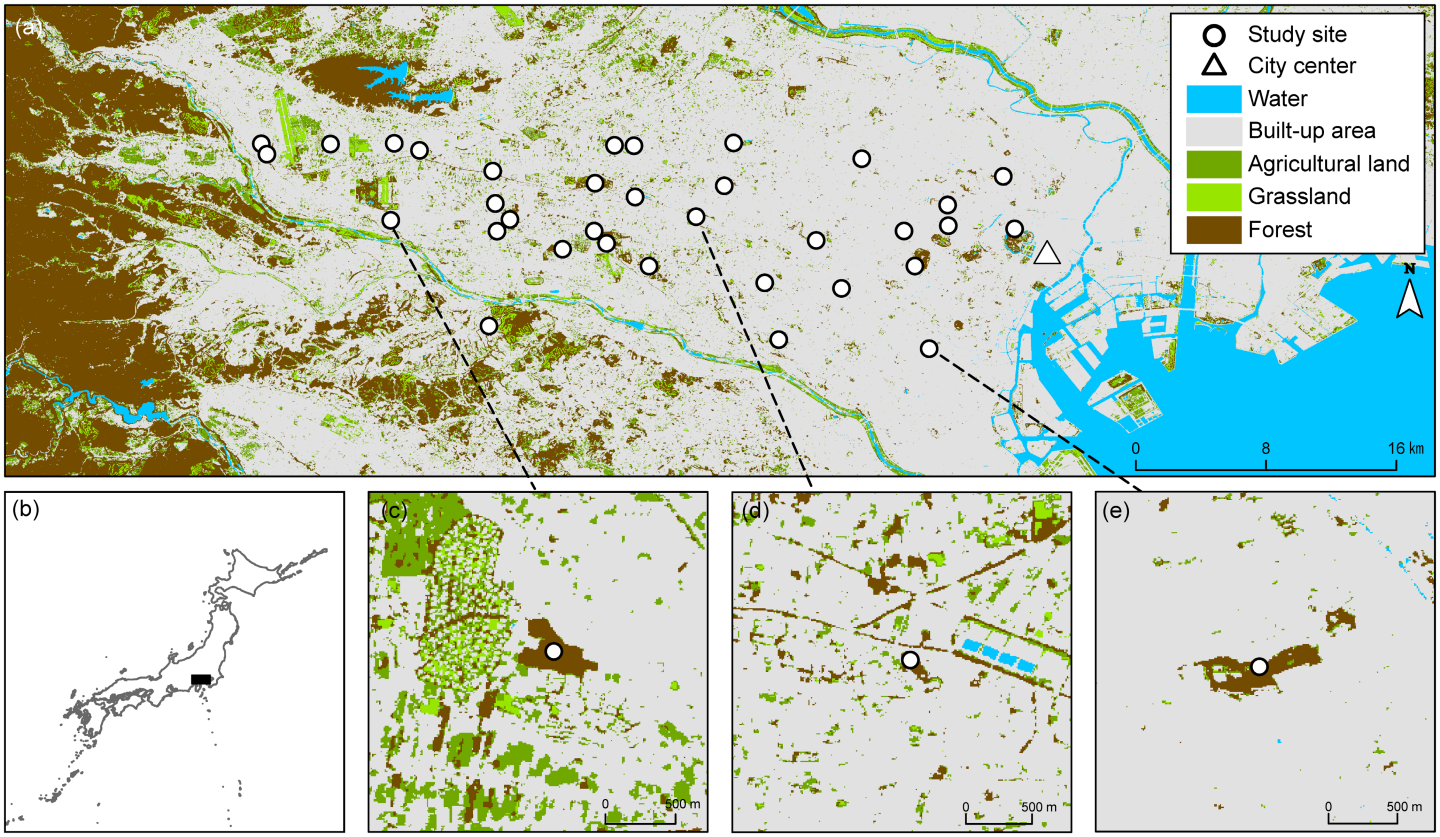
The study area comprises 34 study sites in the Tokyo metropolitan area, Japan. Map showing (a) the location of the study site along the urban–rural gradient, and (b) the location (black square) of the study area in Japan. The surroundings of each site with (c) low, (d) medium, and (e) high urbanization rates. Study sites are shown as white circles, and the city center (Tokyo station) is represented by a white triangle.

This study was conducted in the Tokyo major metropolitan area (Tokyo MMA; 35°39′–36°02′ N, 139°19′–56′ E) (Figure 1), which is the world's largest city, with an agglomeration of 37 million inhabitants (United Nations, 2019). Since the 1600s, this area has undergone remarkable urbanization and population growth, with a simultaneous decline in the extent of natural areas. Although many habitats have been lost and fragmented due to urbanization, this region has also preserved various fragmented habitats. Thus, the Tokyo MMA is composed of mosaic landscapes (agricultural lands, forests, grasslands, and urban areas), with the Kanto Plain having an elevation of 25–142 m above the mean sea level. Furthermore, to prevent the decrease in green space due to urbanization, an increase in the development of green spaces, such as parks, has occurred, thus diversifying the urban green space environment.

The fragmented urban green space comprises various environments, including remnant forests, abandoned forests, and established green parks from diverse environments. These remnant forests consist of secondary forests (dominated by *Quercus serrata* and *Quercus acutissima*) that provide a bright understory to grow young trees and herbaceous plants due to periodic human management activities, such as tree logging (every 10–25 years). In contrast, the abandoned forests (dominated by *Quercus myrsinifolia* and *Aucuba japonica*) comprise dark understory and old trees. After the 1970s, the number of abandoned forests increased due to a decline in the demand for timber, primarily in response to lifestyle changes (Iwachido et al., 2020; Takeuchi et al., 2003). The green parks comprise short grasslands (dominated by *Plantago asiatica, Trifolium repens*, and *Digitaria ciliaris*) and secondary forests that are managed by the administration and residents.

### 2.2 Sampling design and plant survey

Thirty‐four study sites were selected from fragmented large green spaces (>5 ha), along an urban–rural gradient based on the urbanization rate around each site within 1 km. Urbanization rates were calculated using a high‐resolution land cover map with a resolution of 10 m (JAXA, 2020) and ArcGIS Pro 2.5. The map was produced using the average land cover from 2018 to 2020. The urbanization rate for each site ranged from 48.1% to 98.1% (Figure 1).

We established several 20‐m transects at each site, and three plots (1 m^2^) were set on each transect at regular intervals. The number of transects at each study site varied based on the area (areas <1 ha: 4 transects; 1–5 ha: 5 transects; 5–10 ha: 6 transects; 10–30 ha: 7 transects; 30–50 ha: 8 transects; 50–70 ha: 9 transects; > 80 ha: 10 transects). Furthermore, considering that most sites contained forest and grassland habitats, the number of transects at each habitat was determined by the percentage of each habitat size. Given that plant communities may differ within a study site, we set the transects at even intervals to analyze the entire plant community at each study site. Transects were placed in areas of the study site where there was spontaneous growth of many plant species. As a result, 162 transects were surveyed in forests and 55 transects in grasslands.

In 2020, we surveyed the plant species at 651 plots in the summer (June–July) and autumn (September–October), respectively. At each plot, we divided a plot into nine grids and recorded the number of grids where plants appeared on the ground, including herbaceous and woody species; this was defined as the abundance of each plant species in each plot. In subsequent analyses, we divided the plant species into two categories: native and exotic; exotic species were defined as those that did not inhabit Japan before 1868 (NIES, 2002).

### 2.3 Environmental variables

We measured five local factors (soil moisture, management intensity, openness of tree layers (openness), basal area of trees (basal area), and years since the establishment of the habitat (years since establishment)) and three landscape factors (habitat size, the surrounding land use, and spatial structure of habitats) (Table S1).

The soil moisture was measured using a soil moisture meter (DIK‐311F: Daiki Rika Kogyo, Saitama, Japan) three times per plot (used averaged values at each transect in the analyses), the openness was observed using photos acquired by a fisheye lens camera (PIXPRO SP360, Kodak, NY, USA) per transect to assess light conditions in the forest, and the basal areas were recorded within a radius of 3 m from the transect. The management intensity at each transect was based on interviews with park managers and residents. The years since the establishment at each transect were evaluated using aerial photographs from multiple years (1936–2020). The details of the local environment variables are provided in Table S1.

As the landscape factor, we calculated the size of each site and the area of each land use type (agricultural lands, grassland, forest, and urban) surrounding each site. We calculated the area of surrounding land use at three different buffer radii (500, 1000, and 2000 m) which are commonly used as impacts on biological communities (e.g., Brice et al., 2016; Lopez et al., 2018; Perry et al., 2020) but identified the correlations between each land use type for all three buffer radii (*r* > .7; Figure S1); only land use data for the 1000 m buffer size were used in the subsequent analyses. As we calculated the areas of each land use type from the edges of the site, the buffer areas differed; therefore, we applied the percentage of areas occupied by land use types within a buffer. Additionally, we calculated Moran's eigenvector maps (MEMs) (Dray et al., 2006) based on the geographic coordinates of each transect as the spatial distribution of habitats. MEMs produce a set of orthogonal spatial variables that can be used as explanatory variables to indicate multiscale spatial relationships in a community assembly. They are generated by the diagonalization of a doubly centered spatial weighting matrix (SWM) (Bauman, Drouet, Dray, & Vleminckx, 2018a). The SWM was constructed using the Hadamard product of connectivity by a weighting matrix (Dray et al., 2006). Since many variables exist in the connectivity and weighting matrix, selecting SWM is essential to accurately estimate MEMs (Bauman, Drouet, Fortin, & Dray, 2018b; Dray et al., 2006). We performed forward selection with a double‐stopping criterion (FWD; Blanchet et al., 2008) to select an optimal SWM derived from four combinations of two connectivities (Gabriel and minimum planar graphs) and two weighting matrices (concave‐down and concave‐up). FWD can be used to accurately calculate MEMs, with a lower type I error, compared with other variable selection methods (e.g., minimizing the Akaike information criterion and minimizing the autocorrelation) (Bauman, Drouet, Dray, & Vleminckx, 2018a). Then, we estimated the MEM variables displaying positive spatial autocorrelation from an optimal SWM. Since a positive correlation for the spatial autocorrelation of MEM is easy to interpret (the further the distance between sites, the larger its value), this method is often used in multivariate regression and canonical analysis.

### 2.4 Extraction of landscapes with different urbanization rate

To continuously assess the relative importance of local and landscape factors along the urban–rural gradients, we clipped landscapes to contain multiple sites having different urbanization rates using randomly generated buffers. To create these landscapes, we first created a minimum convex hull that included the center of all sites. Then, 100 buffers were randomly generated so that the center of the buffer was included in a convex hull. This approach was repeated 100 times to minimize spatial bias. As the strength of effects on biological communities varied with the spatial scale of sampling (Meynard et al., 2013; Viana & Chase, 2019), four sizes of buffers were created, ranging from 6 km (the maximum distance between adjacent sites) to 9 km in radius. This method was performed separately for habitats (forests and grasslands) and buffer sizes (6–9 km). In this way, eight sets (two habitats × four buffer sizes) of 10,000 buffers (100 buffers × 100 repetitions) were created. Within each set, we removed the buffers having duplicate combinations of sites or those that did not contain more than four sites. Therefore, we created a minimum of 3150 (6000 m buffer size at grassland habitats) and a maximum 6729 (9000 m buffer size at forest habitats) unique site combination (Table S2). In each buffer, using plants, local environment variables, the size of the study site, the proportion of each land use area, and MEM, we estimated the relative importance at local and landscape factor (detail in Section 2.5). Plant data were aggregated per transect, and the abundance and local environment variables were averaged by season. In addition, we calculated the average urbanization rate within each buffer as the urbanization rate.

### 2.5 Statistical analyses

All statistical analyses were performed separately for species (native and exotic), habitats (forest and grassland), and buffer sizes (6–9 km). To estimate the relative importance of local and landscape factors at each buffer, we first identified significant variations that explained the plant species communities at each factor. Variation selection at each factor was performed by the forward selection method using a redundancy analysis (RDA) based on the adjusted coefficient of determination ($R_{\mathrm{adj}}^{2}$) of the global model (Blanchet et al., 2008). RDA combines regression and principal component analysis (PCA) (Borcard et al., 2011). In our study, the response variables (the plant species community) were Hellinger‐transformed, divided by the total abundance at each transect, and then their square root was calculated, dampening the effect of highly abundant species. This transformation has previously been shown to be desirable for RDA (Legendre & Gallagher, 2001). The explanatory variables (all environmental variables) were standardized, and several values with a variance inflation factor (VIF) < 10 were applied. Openness and basal area were not used as local factors for the grassland habitats.

Additionally, we performed variation partitioning based on RDA, using the selected explanatory variables in the forward selection method. Variation partitioning can quantify the relative importance of explanatory variables by controlling the effects of other variables (Borcard et al., 2011). We used $R_{\mathrm{adj}}^{2}$ to reflect the relative importance of each factor (purely explained by each factor), which considered the number of explanatory variables and sample sizes to prevent inflation of the *R* ^2^ values (Peres‐Neto et al., 2006). If the forward selection selected no explanatory variables, the relative importance was set to zero. For exotic species in grassland habitats at 6000 m buffer, most landscape variables were not selected through forward selection methods, therefore, their relative importance was not estimated.

To compare the relative importance of each factor across the urban–rural gradient, we used a LMM with relative importance of each factor as the response variables, category of factor (local or landscape) as explanatory variables, repetition identity and buffer identity as random effects. The relationships between the relative importance of each factor and the urbanization rate were tested using the LMM. This model used the relative importance of each factor (local and landscape) as the response variable, the average urbanization rate within each buffer as the explanatory variable, repetition identity as the random intercept, and the average urbanization rate as the random slope. Finally, to estimate the relative importance of each environmental variable selected by the forward selection method, we performed a permutation test for the joint effects of constraints in RDA using *anova.cca* function in vegan package (Oksanen et al., 2020). To test the relationships between the relative importance of each environmental variable and the urbanization rate, we used LMM with the relative importance of each environmental variable as the response variables, average urbanization rate within each buffer as the explanatory variable, and repetition identity as random effects.

All analyses were carried out using R version 4.02 (R Development Core Team, 2022), with *adespatial* 0.3–14 (Dray et al., 2021), *lme4* 1.1–31 (Bates et al., 2022), and *tidyverse* 2.0.0 (Wickham et al., 2019) packages.

## 3 RESULTS

A total of 438 plant species were identified across the 34 sites, including 353 native and 85 exotic species. The species richness was 412 (native: 339, exotic: 73) in forest habitats and 157 (native: 111, exotic: 46) in grassland habitats.

**FIGURE 2:**
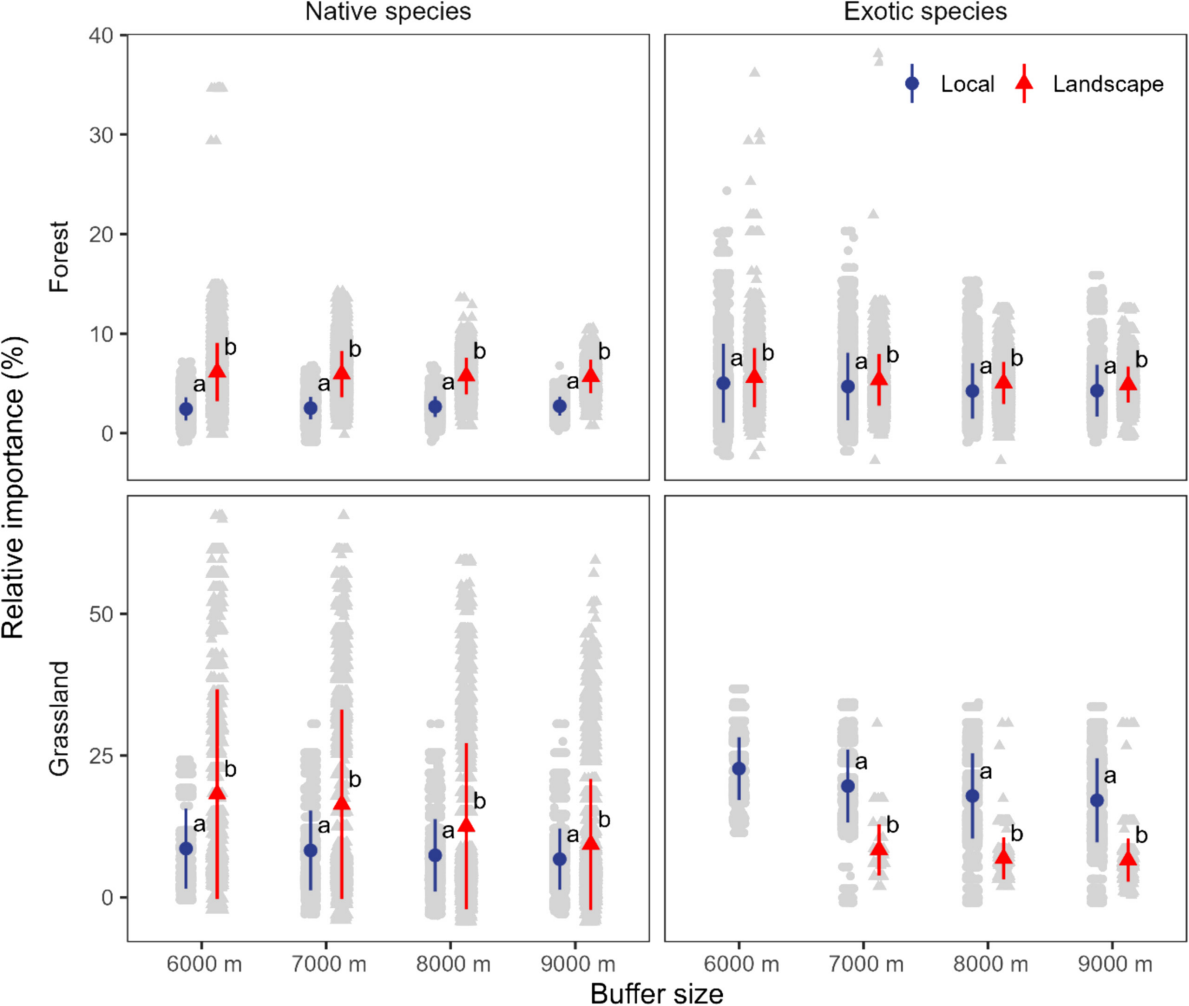
Comparison of the relative importance of local and landscape factors at each buffer size. The colored dots and lines indicate the mean and standard deviation, respectively. The upper and lower panels portray forest and grassland habitats, respectively. The left and right panels portray native and exotic species, respectively. Different letters indicate significant differences based on the linear mixed model at each buffer size.

At maximum, we could explain the 32% and 33% variation in plant species composition in forest habitats and 48% and 38% in grassland habitats for native and exotic species, respectively. Across urban–rural gradients, the relative importance of landscape factor was significantly higher than that of local factor, except for exotic species in grassland habitats (Figure 2).

**FIGURE 3:**
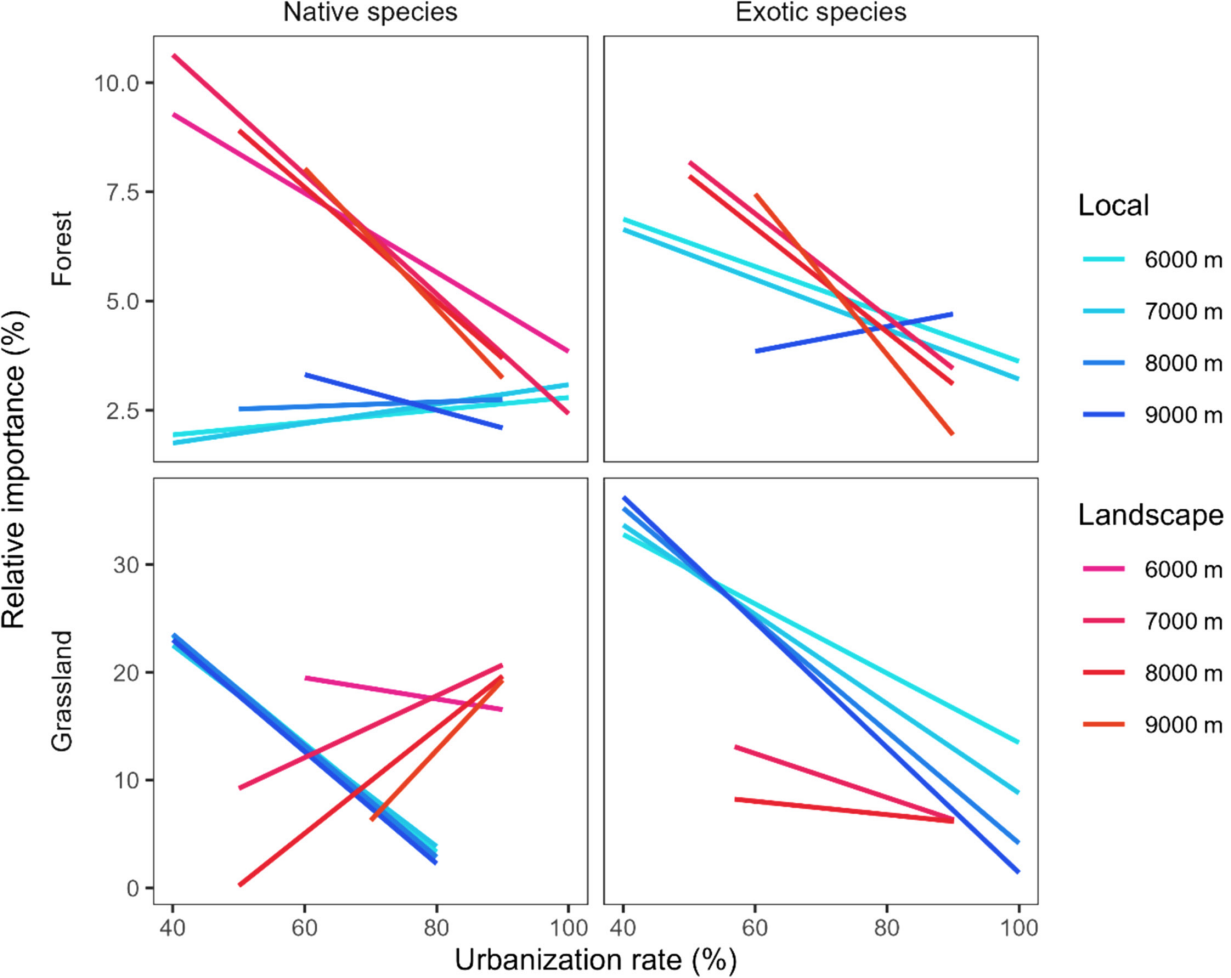
Relationships between the relative importance of local and landscape factors and urbanization rate at each buffer size (from 6000 m to 9000 m), based on the linear mixed model. The upper and lower panels portray forest and grassland habitats, respectively. The left and right panels portray the native species and exotic species, respectively. Colors indicate each buffer size at each factor (local and landscape). The solid line indicates that the relationships were significant (*p*‐value < .05).

The relationships between the relative importance of local and landscape factors varied with the urbanization rate (Figure 3; Figures S2 and S3). Specifically, the relative importance of local factors for grassland habitats significantly decreased with the urbanization rate, regardless of species (Figure 3; Figure S2). However, in forest habitats, the urbanization impact on the relative importance of local factors varied by species. For exotic species, the relative importance of local factor at the 6000 m and 7000 m buffers decreased with the urbanization rate, while for native species, this increase was observed at 6000 m, 7000 m, and 8000 m buffers. At the 9000 m buffer, the relationship between the relative importance of local factors and urbanization rate exhibited opposite trends compared with other buffer sizes for each species in forest habitats. There was no relationship between the relative importance of local factor and urbanization rate, for exotic species in forest habitats at the 8000 m buffer. Additionally, the changes in the relative importance of landscape factors due to urbanization rate varied by habitats. In forest habitats, the relative importance of landscape factor decreased with the urbanization rate, whereas the opposite trend was observed in grassland habitats (Figure 3; Figure S3). However, in grassland habitats, the impacts of urbanization on the relative importance of landscape factor depended on the species. While the relative importance of landscape factor increased with urbanization rate for native species, it decreased for exotic species. The relative importance of the landscape factor for exotic species at the 6000 m buffer in forest habitats, as well as at the 7000 m and 9000 m buffers for exotic species, remained unchanged with the urbanization rate (Figure 3; Figure S3). The residuals (percentage of unexplained by variation partitioning) increased with the urbanization rate (Figure S4). However, there was no relationship between the residuals and urbanization rate at the 8000 m buffer for native species in grassland habitats.

In local factors, the years since establishment and management intensity were the main factors, and their relative importance varied with urbanization rate (Figure S5). While the relative importance of years since establishment increased with urbanization rate, that of management intensity decreased. The relative importance of basal area and openness decreased with urbanization rate at 6000 m, 7000 m, and 8000 m buffer in forest habitats for exotic species. In landscape factors, MEM was the main factor, and their relative importance increased with urbanization rate in forest habitats and for exotic species in grassland habitats, while it decreased for native species in forest habitats (Figure S6). In forest habitats, the relative importance of the agricultural rate only decreased with the urbanization rate, and the relative importance of other landscape factors increased. In grassland habitats, the urbanization impacts on the relative importance of each landscape factor depended on buffer size.

## 4 DISCUSSION

This study reveals that the relative importance of the landscape factor was higher than that of the local factor across urban–rural gradients. However, the relative importance of local and landscape factors varied along the urban–rural gradient, and their trends depended on habitats and species. In forest habitats, while the relative importance of landscape factor decreased with urbanization rate, whereas that of local factor increased for native species. Conversely, in grassland habitats, the relative importance of the local factors decreased with urbanization rate, whereas that of landscape factors increased for native species. To the best of our knowledge, this study represents the first evidence of shifts in the relative importance of factors influencing plant communities along urban–rural gradients.

Consistent with our hypothesis, the relative importance of local factors increased with urbanization rate for native species in forest habitats. Furthermore, among the local factors, the relative importance of years since establishment was highest for native species in forest habitat and increased with urbanization rate. This outcome may reflect the increase in environmental heterogeneity associated with urbanization (McKinney, 2006). As urbanization has led to the reduction in the remnant green space, attempts have been made to mitigate the loss of green space by developing new ones (Dearborn & Kark, 2010). Consequently, the city center contains green space with diverse histories, ranging from remnants to newly developed green spaces. This landscape gives rise to a significant degree of environmental heterogeneity within and among urban green spaces (Aronson et al., 2017). Indeed, the history of green spaces has modified the habitat environment, creating a habitat for a variety of plant species in urban ecosystems (Iwachido et al., 2023; Wallace et al., 2017). Hence, urbanization contributes to an increase in environmental heterogeneity, which in turn enhances the impact of environmental filtering (Kraft et al., 2015). Moreover, similar positive relationships between the relative importance of local factors and environmental heterogeneity have been reported in the studies of mammal communities in tropical rainforests (Wearn et al., 2019).

Furthermore, consistent with our hypothesis that changes in the relative importance of local factors due to urbanization depend on species. In forest habitats, the relative importance of local factors increased for native species, whereas it decreased for exotic species. In particular, among the local factors influencing exotic species in forest habitats, the relative importance of openness, basal area, and management intensity decreased with urbanization rate. This underscores the profound impacts of changing human‐nature interactions in rural areas. In recent years, the abandonment of secondary forests, prevalent in this region, has accelerated due to declining and aging populations and shifting lifestyles (Takeuchi et al., 2003). These abandoned secondary forests have been observed to darken the forest understory and induce substantial changes in the plant community composition (Iwachido et al., 2020; Uchida et al., 2023). Given the preference of exotic species for bright environments, the increasing of abandoned secondary forests in rural areas, which are less invasive to exotic species, may increase the relative importance of the local factor in rural areas. Similarly, in grassland habitats, the relative importance of local factors decreased with urbanization rate, particularly decreased in the relative importance of management intensity. The urban grasslands primarily comprise developed green spaces for recreational purposes. In these green spaces, annual forbs and wind dispersal plant species dominate due to the intense mowing to make them accessible to many people. Thus, similarities in management practices may have led to increased environmental similarities in urban grasslands (Polsky et al., 2014), as most green spaces are managed for safety.

As we hypothesized, the relative importance of landscape factors decreased with urbanization rate. This trend likely stems from a reduction in the environmental heterogeneity surrounding green spaces due to urbanization. Generally, urbanization leads to a homogenization of green space use patterns (McKinney, 2006), which diminishes the variability of the surrounding environment and potentially reduces the relative importance of landscape factors by limiting the introduction of species from diverse surrounding areas. Specifically, the decreasing the relative importance of agricultural rate around green spaces with urbanization rate for native species in forest habitats may highlight the significant impacts of the agricultural land. Given that the green spaces targeted in this study were mainly parks and forests, the presence of agricultural land with distinct plant species compositions likely influences the native plant species. Conversely, while the relative importance of landscape factor increased with the urbanization rate for native species in grassland habitats, the relationship between the relative importance of each landscape factor and urbanization rate varied with buffer size. This suggests that it is the diversity of green spaces rather than any specific green spaces that affect native plant species in grassland habitats (Deutschewitz et al., 2003). However, our study did not account for the heterogeneity of the surrounding environment, warranting future studies on the effects on plant communities.

Importantly, we were only able to account for approximately half of the variation in the plant community as we have not yet quantified all the environmental changes affecting plant communities associated with urbanization. Moreover, we found that residuals increased with the urbanization rate. This indicates that factors not observed in this study have a stronger impact on plant communities in the urban center. Since most of the surveyed sites are parks, trampling by people will likely affect the plant communities. In fact, trampling experiments show that its effect on plant communities varies with the intensity and duration of the trampling (Rusterholz et al., 2021). In particular, grasslands are used by many people and will most likely be heavily impacted by humans. Furthermore, in habitats that are heavily used by humans, it can be expected that there will be significant impacts from exotic species. Previous studies have shown that urbanization increases exotic species (Aronson et al., 2015; Cadotte et al., 2017). Thus, the increased effects of interspecific competition in plant communities may be expected as competitive species are expected to dominate among exotic species with progressing urbanization (Cadotte et al., 2017). In recent years, there has been an increase in the number of studies on phenotypic variations within populations (Thompson et al., 2022) and species evolution (Johnson & Munshi‐South, 2017; Lambert & Donihue, 2020) due to urbanization. Therefore, to accurately identify the factors that affect species communities in urban areas, it is imperative to evaluate these factors, including evolutionary and competitive processes.

## 5 CONCLUSIONS

The process of rapid urbanization has exerted substantial impacts on ecosystems by habitat fragmentation and degradation. However, the magnitude and direction of these effects have been dependent on the specific context. In our study, we have demonstrated the relative importance of local and landscape factors influencing plant communities' changes in response to varying urbanization rate. This suggested that previous studies conducted in areas with different environments, such as urbanization gradients, have not been able to accurately assess the factors influencing biological communities. Hence, to advance the general understanding of the impacts of urbanization, it will be essential in the future to elucidate not only the patterns but also the underlying mechanisms by which factors change. Furthermore, since the impact of urbanization varies by city size and habitat, it is essential that future research must be conducted in multiple cities across Asia and Africa, where urban studies have been relatively limited.

## AUTHOR CONTRIBUTIONS

**Yuki Iwachido:** Formal analysis (lead); funding acquisition (equal); investigation (lead); writing – original draft (lead); writing – review and editing (equal). **Kei Uchida:** Funding acquisition (equal); investigation (equal); writing – review and editing (equal). **Takehiro Sasaki:** Funding acquisition (equal); methodology (supporting); writing – review and editing (equal).

## CONFLICT OF INTEREST STATEMENT

We declare that they have no conflicts of interest.

## Supporting information


Appendix S1

## Data Availability

All code and datasets used in the analysis are available at the following online repository (*figshare*) (https://doi.org/10.6084/m9.figshare.19164680.v2).
