# Supplementary material for: Urbanization alters the relative importance of local and landscape factors affecting plant communities in the Tokyo megacity
Source: Ecol Evol. 2024 Aug 29;14(9):e70006. doi: 10.1002/ece3.70006 (PMC11361761; doi:10.1002/ece3.70006)
Supplement: Supplementary file 1 — Appendix S1 [file ECE3-14-e70006-s001.docx]

| **Explanatory variables** | |  | **Details** |
| --- | --- | --- | --- |
| *Local factor* | |  |  |
|  | Soil moisture (%) |  | Average soil moisture among the three plots in each line. Using DIK-311F (Daiki Rika Kogyo, Saitama, Japan), three measurements were taken at each plot. The measurement was taken at least two days after an intense rainfall (>30 mm day^−1^). |
|  | Years since establishment |  | It was assessed through observations of multitemporal aerial photographs (1936–2020). Aerial images were obtained from the Geospatial Information Authority of Japan. Because the time intervals of aerial photographs for the entire periods at all study sites varied, the years since establishment were rounded. |
|  | Management intensity |  | Based on interviews and field surveys, the management intensity at each line was evaluated on three scales:  *Forest*   1. Abandoned: Mowing has not been done or has been done every few years, dominated plants are bamboo (such as *Pleioblastus chino*) and evergreen broad-leaved trees (such as *Quercus myrsinifolia*). The understory plant height is more than 1 m. 2. Intermediate-managed: Mowing 2-3 times a year, dominated plant area forbs. The understory plant height is 0.3–0.7 m. 3. Heavily managed: Due to trampling and intensive management (mowing at least four times a year), the understory plant height is less than 30 cm (dominant by *Plantago asiatica* and *Hydrocotyle sibthorpioides*).   *Grassland*   1. Low-managed: Mowing once a year, dominated plants are taller than 0.8 m (such as *Miscanthus sinensis* and *Imperata cylindrica*). 2. Intermediate-managed: Mowing 2-3 times a year, dominated plants are broad-leaved herbs, and height is 0.3–0.7 m. 3. Heavily managed: Due to trampling and intensive mowing at least four times a year and plant height less than 0.3 m (such as *Trifolium repens* and *Digitaria ciliaris*) |
|  | Canopy openness (%) |  | It is calculated from hemispherical photographs captured in the center of each line. The photos were taken using a fisheye lens camera (PIXPRO SP360: Kodak, NY, USA), and the openness was calculated using CanopOn2 (http://takenaka-akio.org/etc/canopon2/). |
|  | Basal area (m²) |  | The total basal area for trees was measured, with a diameter at breast height (DBH) >5 cm within a 3-m radius from each line. |
|  |  |  |  |
| *Landscape factor* | |  |  |
|  | Site area (km²) |  | Area of each site. |
|  | Agricultural land (%) |  | The area ratio of each land use type within 1000 m from each site. |
|  | Grassland (%) |  |  |
|  | Forest (%) |  |  |
|  | Urban (%) |  |  |
|  | Moran's eigenvector map (MEM) |  | MEM is a multi-scale spatial variable created from the location of each site. For details, see Section 2. |

**Table S1.** Description of each explanatory variable.

| **Habitat** | **Buffer size** | **Number of buffers** | **Number of buffers  at each repetition** | |
| --- | --- | --- | --- | --- |
|  |  |  | Maximum | Minimum |
| Forest | 6000 | 5936 | 72 | 48 |
|  | 7000 | 6711 | 74 | 60 |
|  | 8000 | 6745 | 79 | 57 |
|  | 9000 | 6729 | 75 | 55 |
| Grassland | 6000 | 3150 | 38 | 24 |
|  | 7000 | 4430 | 54 | 36 |
|  | 8000 | 4960 | 56 | 41 |
|  | 9000 | 5026 | 59 | 40 |

**Table S2** Number of buffers at each set and at each repetition.


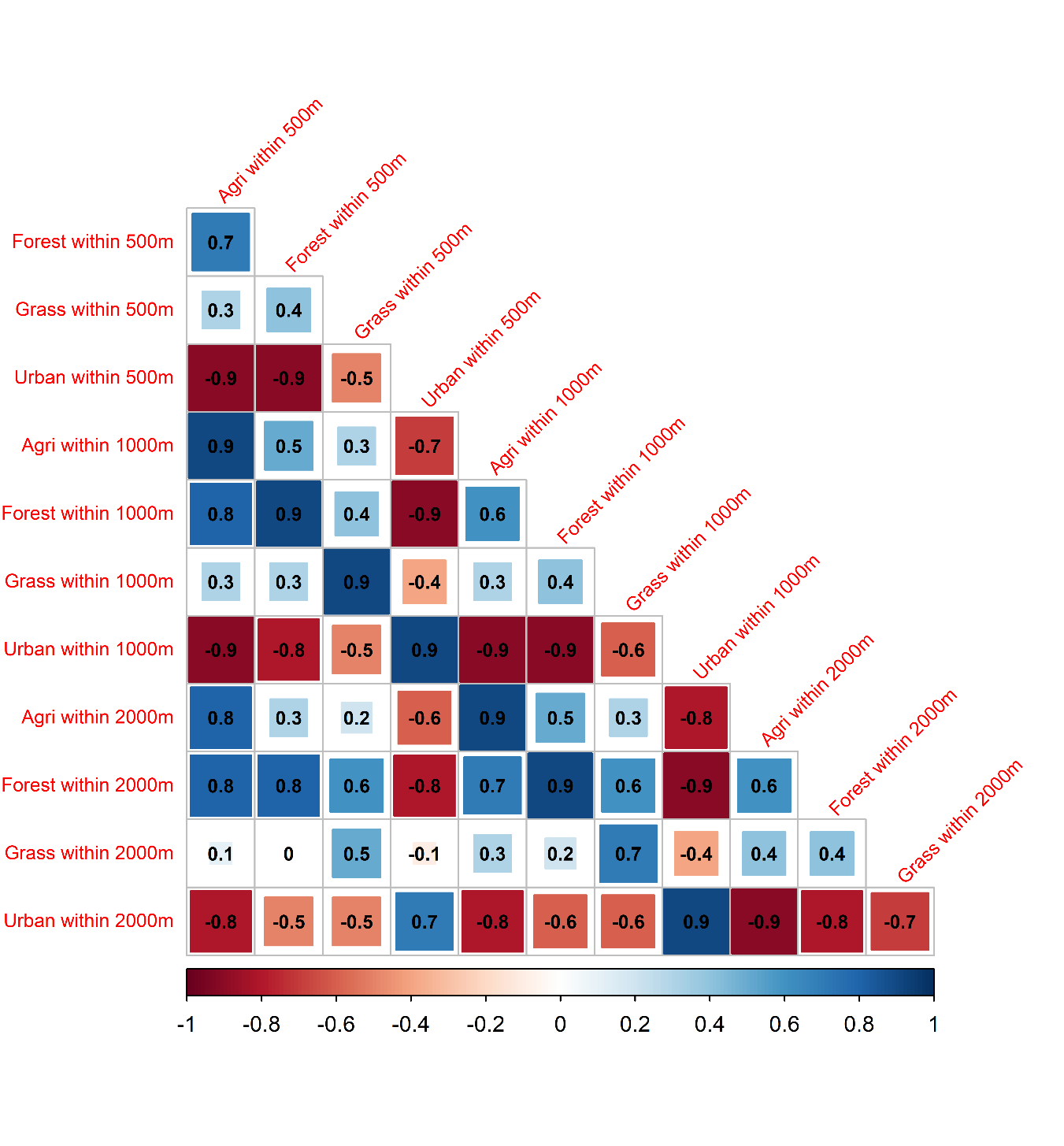
**Fig. S1.** Correlation between all landscape variables. Each value indicates the correlation coefficient between the two variables. The square size indicates the magnitude of the correlation coefficient, and the color of the square indicates the direction of the correlation. The bar is a legend portraying the amount of the correlation coefficient. Agri = agricultural land.


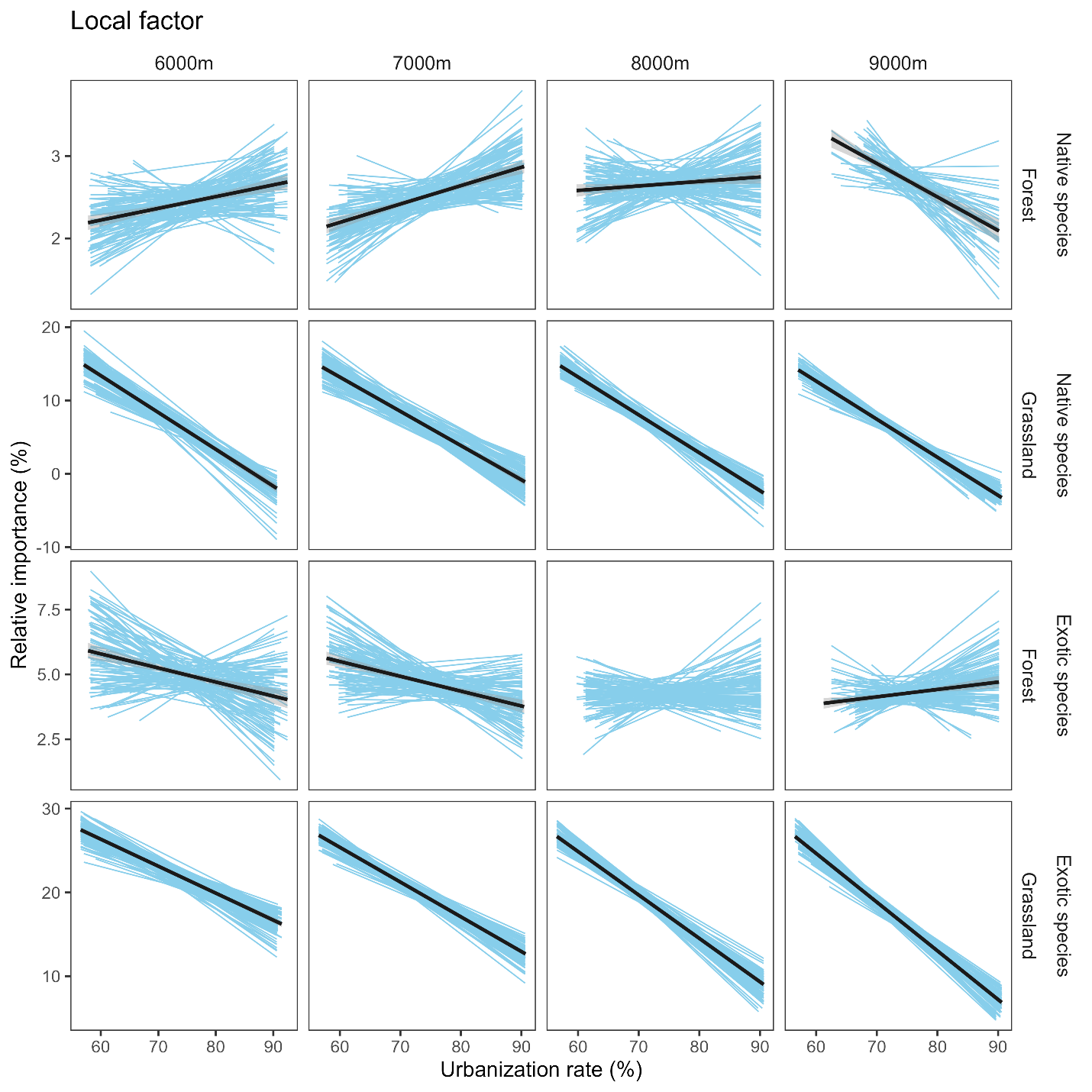


**Fig. S2.** Relationships between the relative importance of local factor and urbanization rate at each buffer size (6000-9000m: each column), habitat (forest and grassland), and species (native and exotic species) based on the linear mixed model. Each column shows each buffer size. Light bule line portray its relationships at each repetition. The black line indicates that the model including all repetitions are significant (p-value < 0.05).


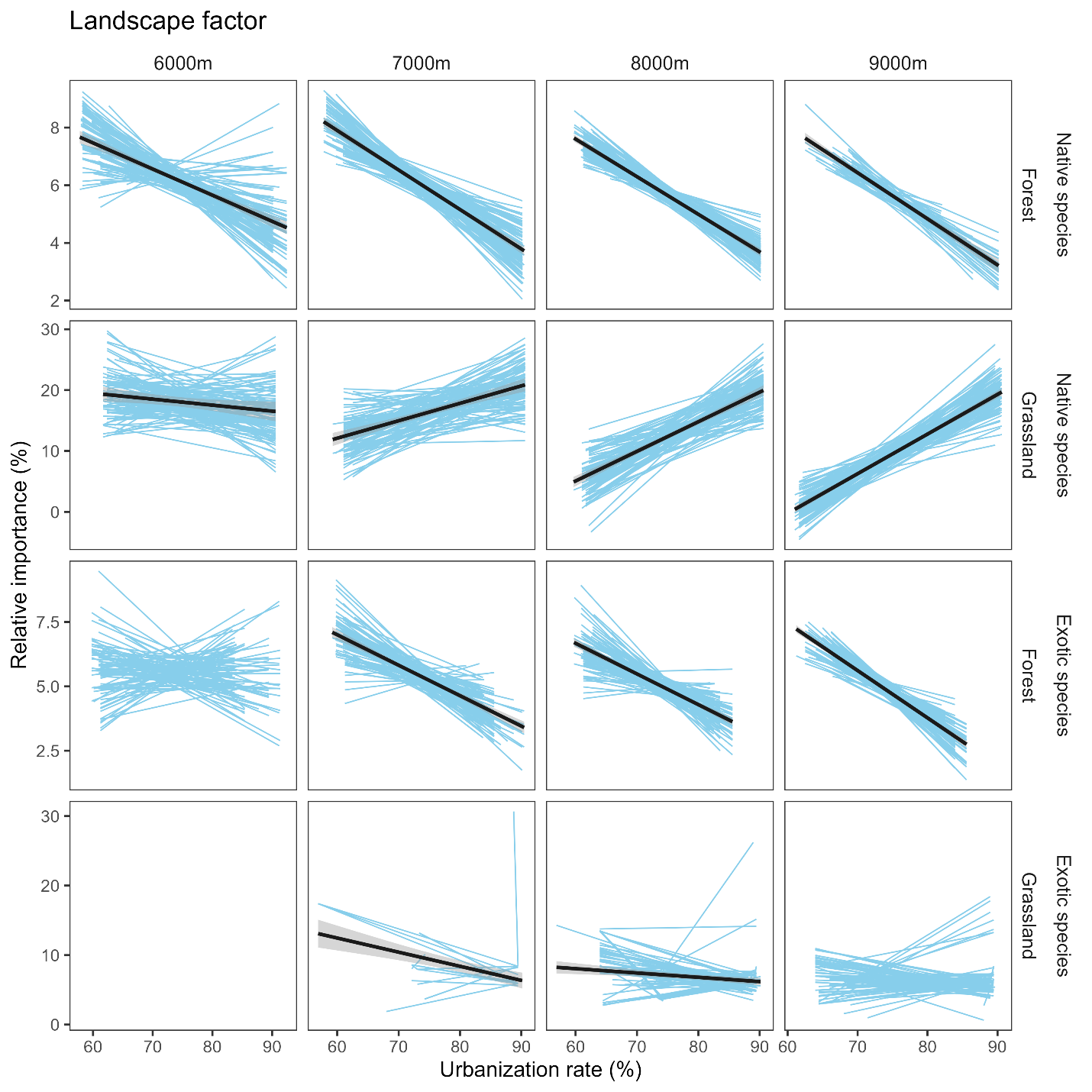


**Fig. S3.** Relationships between the relative importance of landscape factor and urbanization rate at each buffer size (6000-9000m: each column), habitat (forest and grassland), and species (native and exotic species) based on the linear mixed model. Each column shows each buffer size. Light bule line portray its relationships at each repetition. The black line indicates that the model including all repetitions are significant (p-value < 0.05).


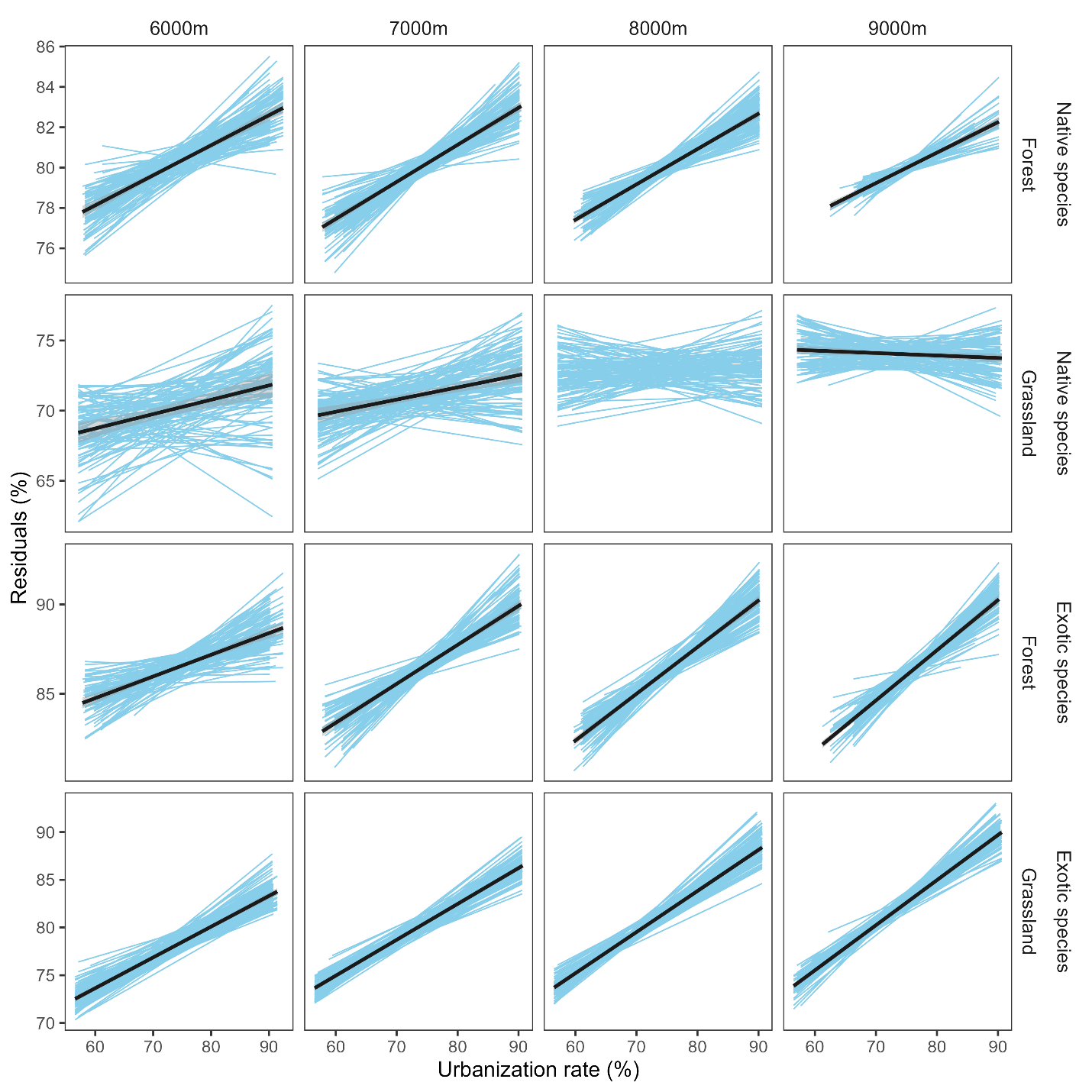


**Fig. S4.** Relationships between the residuals and urbanization rate at each buffer size (6000-9000m: each column), habitat (forest and grassland), and species (native and exotic species) based on the linear mixed model. Each column shows each buffer size. Light bule line portray its relationships at each repetition. The black line indicates that the model including all repetitions are significant (p-value < 0.05).


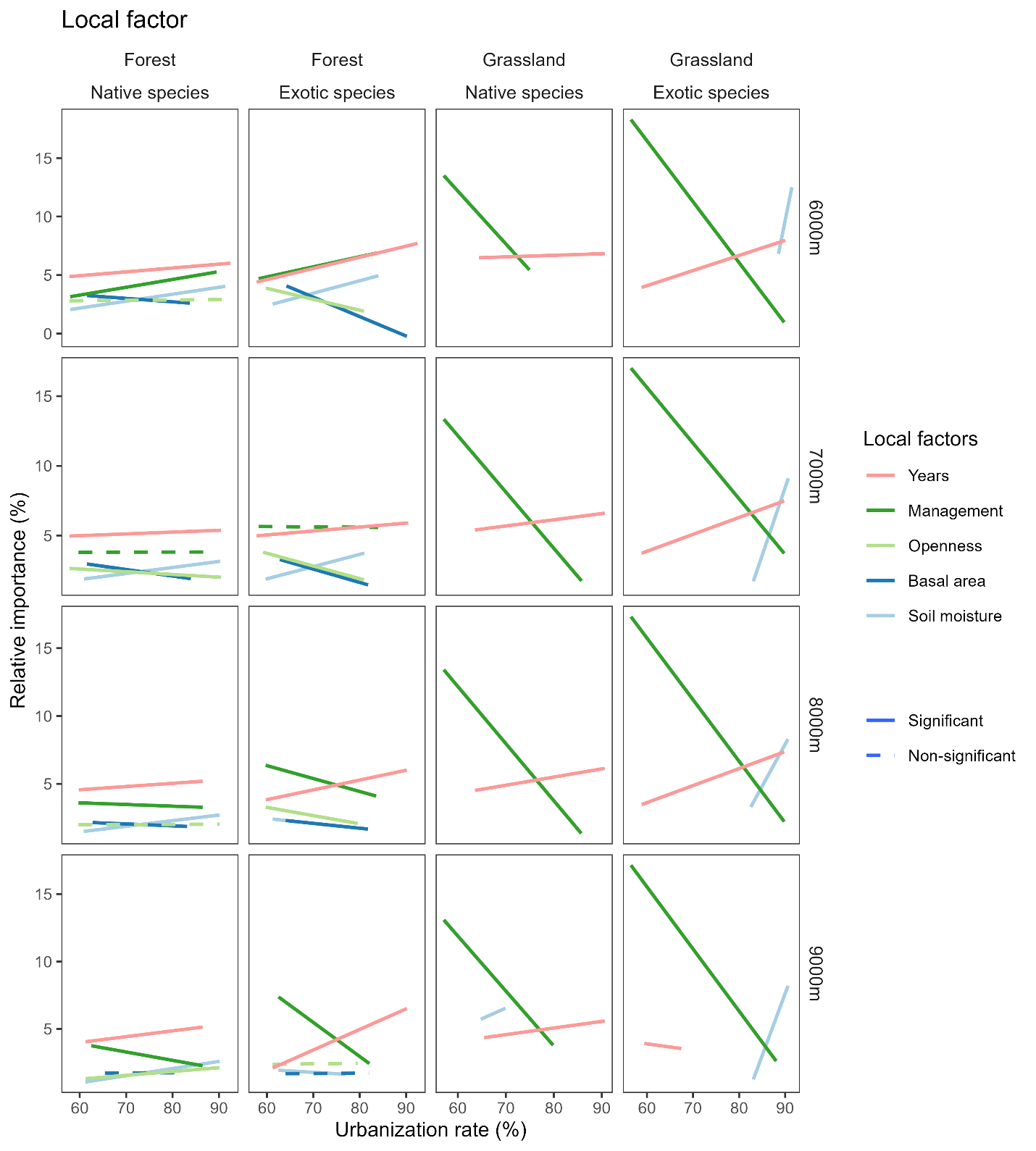
 **Fig. S5.** Relationships between the relative importance of each local factor and urbanization rate at each buffer size (6000-9000m: each column), habitat (forest and grassland), and species (native and exotic species) based on the linear mixed model. The solid line indicates that the model is significant (p-value < 0.05).


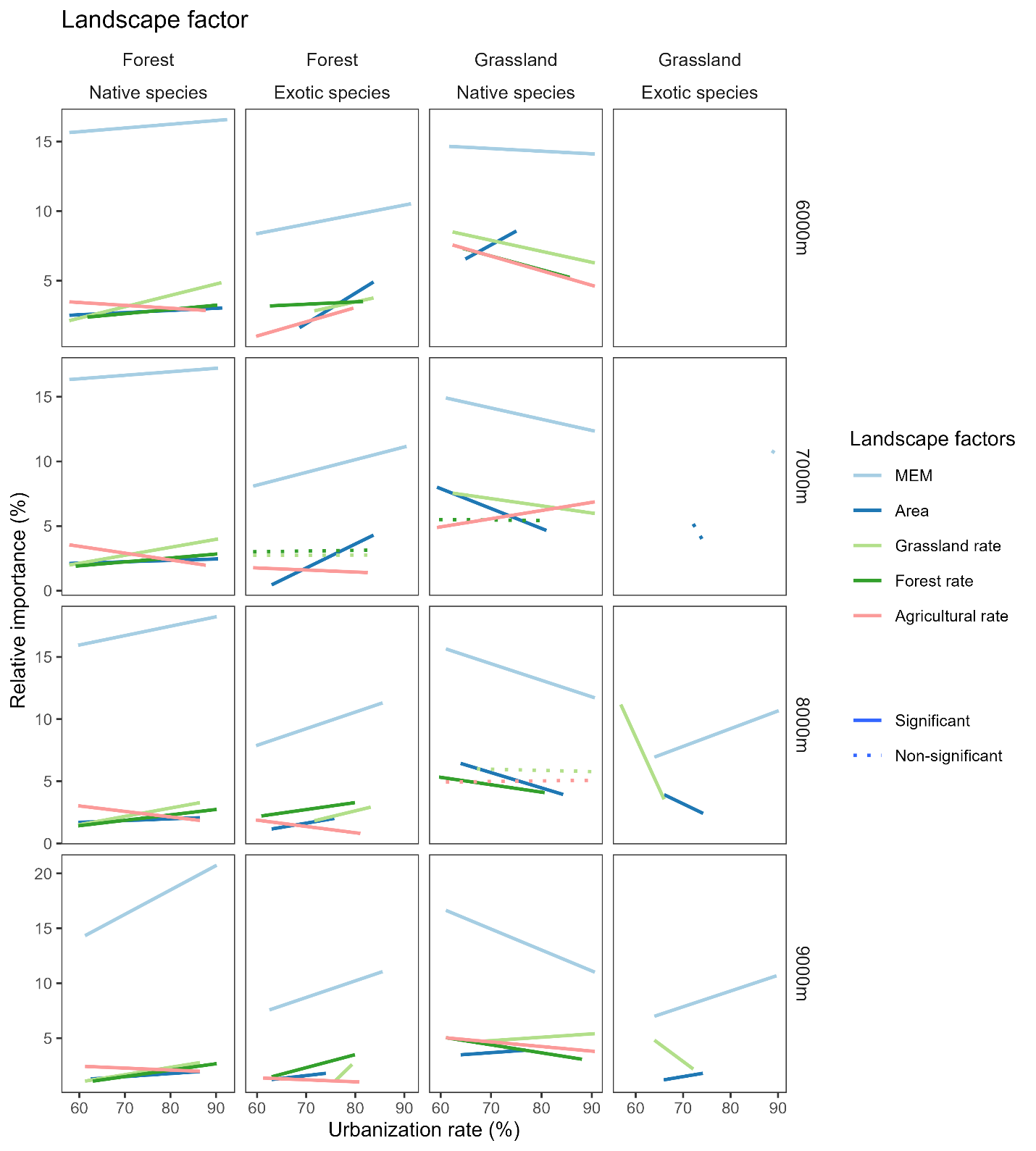


**Fig. S6.** Relationships between the relative importance of each landscape factor and urbanization rate at each buffer size (6000-9000m: each row), habitat (forest and grassland), and species (native and exotic species) based on the linear mixed model. The solid line indicates that the model is significant (p-value < 0.05).
